# Recombinantly expressed rhFEB remodeled the skin defect of db/db mice

**DOI:** 10.1007/s00253-024-13021-9

**Published:** 2024-01-29

**Authors:** Xiaomin Li, Xinliang Mao, Jianhang Cong, Qirong Zhang, Wenjie Chen, Kunjun Yan, Yadong Huang, Dun Su, Qi Xiang

**Affiliations:** 1 Perfect Life and Health Institute Co., Ltd, Zhongshan, China; 2 Institute of Biomedicine and Guangdong Provincial Key Laboratory of Bioengineering Medicine, Jinan University, Guangzhou, China; 3 Biopharmaceutical R&D Center, Jinan University, Guangzhou, China

**Keywords:** rhFEB, M1 macrophages, db/db mice, Wound healing

## Abstract

**Abstract:**

Fibronectin (FN) and collagen are vital components of the extracellular matrix (ECM). These proteins are essential for tissue formation and cell alignment during the wound healing stage. In particular, FN interacts with collagens to activate various intracellular signaling pathways to maintain ECM stability. A novel recombinant extra domain-B fibronectin (EDB-FN)-COL3A1 fusion protein (rhFEB) was designed to mimic the ECM to promote chronic and refractory skin ulcer wound healing. rhFEB significantly enhanced cell adhesion and migration, vascular ring formation, and the production of new collagen I (COL1A1) in vitro. rhFEB decreased M1 macrophages and further modulated the wound microenvironment, which was confirmed by the treatment of db/db mice with rhFEB. Accelerated wound healing was shown during the initial stages in rhFEB-treated db/db mice, as was enhanced follicle regeneration, re-epithelialization, collagen deposition, granulation, inflammation, and angiogenesis. The wound chronicity of diabetic foot ulcers (DFUs) remains the main challenge in current and future treatment. rhFEB may be a candidate molecule for regulating M1 macrophages during DFU healing.

**Key points:**

• *A recombinant protein EDB-FN-collagen III (rhFEB) was highly expressed in Escherichia coli*

• *rhFEB protein induces COL1A1 secretion in human skin fibroblasts*

• *rhFEB protein accelerates diabetic wound healing*

**Supplementary Information:**

The online version contains supplementary material available at 10.1007/s00253-024-13021-9.

## Introduction

The management of chronic wounds, such as diabetic ulcers, is a major societal concern on a global scale. In diabetes, hyperglycemia disrupts the sequential progression of cellular and molecular events in the healing cascade, consequently resulting in delayed wound healing (Sun et al. 2022). Wound healing involves a synergistic effect between endothelial cells, inflammatory cells, macrophages, and keratinocytes (Schreml et al. 2010). The proliferation, migration, and differentiation of the aforementioned cells can trigger inflammatory cell infiltration, angiogenesis, granulation structure formation, collagen deposition, re-epithelialization, and hair follicle development, ultimately promoting wound healing (Hu et al. 2018; Singla et al. 2017). Macrophages, functioning as innate immune cells, play critical roles in wound healing by suppressing inflammatory responses, eliminating cellular debris, and orchestrating tissue repair (Kim and Nair 2019; Okabe and Medzhitov 2016). Typically, nonpathological wounds undergo M1 (inflammatory) to M2 (anti-inflammatory) differentiation approximately 3 days after trauma, with the peak occurring on the seventh day (Louiselle et al. 2021). Conversely, in diabetic wounds, the macrophage phenotype is predominantly M1 (Miao et al. 2012), leading to an observed imbalance and persistent cell polarization (Ganesh and Ramkumar 2020). Therefore, inhibiting the excessive development of M1 macrophages is particularly important for shortening the inflammatory cycle and accelerating wound healing.

The ECM is a platform for the expression of active molecules and for cellular functions, and its remodeling is directly related to the effectiveness of wound healing. FNs and collagen are important bioactive molecules within the ECM that play essential roles in maintaining the structural integrity of the ECM and governing cellular behavior (Chermnykh et al. 2018; Ge et al. 2020). FNs are critical for tissue formation and cellular organization during the wound healing process, including inflammation, proliferation, and remodeling. FNs is usually divided into two types, plasma FNs and cellular FNs, which exhibit different and independent functions in tissue repair processes. (To and Midwood 2011). Plasma FN primarily functions in the initial phase of wound healing by facilitating clot formation and orchestrating the assembly of the ECM. Cellular FN regulates tissue remodeling in the later stages (Mao and Schwarzbauer 2005; Patten and Wang 2021). FN isoforms exhibit greater efficacy in selectively promoting wound healing than dose full-length FN owing to the varying availability of binding domains (Vogel 2018). Increased vascular endothelial growth factor (VEGF), endothelial cell proliferation, and angiogenesis were observed in the presence of EDB-FN (Khan et al. 2005). EDB-FN is being investigated as a promising therapeutic target for promoting cell proliferation to address endothelial dysfunction caused by diabetes (Khan and Chakrabarti 2006; Lemańska-Perek and Adamik 2019). Furthermore, EDB-FN has been implicated in the immune regulatory pathway. The synthesis and secretion of EDB-FN play a role in the phagocytosis of immune cells that target inflammatory factors, with potential enhancement in the presence of integrin (Kraft et al. 2016).

Collagen regeneration and deposition are important criteria for evaluating the efficacy of wound healing (Wu and Chen 2016). In the inflammatory phase, the degradation of collagen can activate immune cells to eliminate necrotic tissue and bacteria (Yeung and Kelly 2021), thereby regulating the extent of the inflammatory response and mitigating the likelihood of wound infection (Sun et al. 2022). The dynamic imbalance between the synthesis and decomposition of collagen is an important factor leading to the slow healing of diabetic wounds in the inflammatory stage (Dinh et al. 2012). In the later stages of wound healing, collagen facilitates the migration, re-epithelization, and deposition of keratinocytes (Chen et al. 2019; Dong et al. 2022) as well as the synthesis of FN in the wound tissue (Lai et al. 2020). Wound healing is accompanied by the conversion of COL3A1 to COL1A1, which is the result of FN recognizing and binding to COL3A1 during the proliferative phase (Beyeler et al. 2019). In addition, COL1A1 promotes angiogenesis and is important for elastic formation after skin repair (Fisher et al. 2009; Reilly and Lozano 2021).

The investigation of wound dressings and the exploration of efficacious strategies to enhance wound healing and mitigate inflammatory responses are highly important for addressing chronic and refractory skin ulcer wound healing. In this study, the active fragment of cellular FN and EDB-FN, and the triple-helix fragment of COL3A1, which is involved in cell adhesion, were carefully selected, and a novel recombinant human FN, referred to as rhFEB, was successfully obtained using recombination technology. The therapeutic efficacy of rhFEB on chronic and refractory skin ulcer healing was evaluated using a full-thickness skin defect model in db/db mice. The findings of this research show good potential for advancing the development of novel functional biomaterials that are based on FN and collagen.

## Materials and methods

### Materials

Vectors pET-20b (Invitrogen, Guangzhou, China) and *E. coli* BL21(DE3) (Invitrogen, Guangzhou, China, ATCC® BAA-1025TM) were used for cloning and heterologous expression. HaCaT (Human Immortalised Keratinocytes, Chinese Academy of Sciences, Shanghai, China, ATCC® no.BNCC101683) were cultured in H-DMEM medium (Gibco, Carlsbad, CA, USA) containing 10% fetal bovine serum (FBS). HSF (human fibroblasts, Guangzhou Saiye Biotech Co., Ltd, ATCC® PCS-200–011) were cultured in DMEM-F12 (Gibco, Carlsbad, CA, USA) containing 10% FBS. ECV304-eGFP (human umbilical vein endothelial cells–enhanced green fluorescent protein, Chinese Academy of Sciences, Shanghai, China, ATCC® PCS-100–010) were cultured in RPMI 1640 medium (Gibco, Carlsbad, CA, USA) containing 10% FBS. Male C57BL/6 mice (6–8 weeks, 20–25 g) purchased from the Guangdong Medical Laboratory Animal Centre (Guangdong, China, certificate no. 44007200069979).

### Expression and purification of rhFEB protein in *E. coli*

Based on the nucleic acid sequences of human EDB-FN (ID: AB191261.1, 4596–4902 nt) and COL3A1 (ID: BC028178.1, 1562-1651nt) in the GeneBank database, the cDNA sequence of recombinant human EDB-FN-collagen III (rhFEB) was acquired. The cDNA sequence of rhFEB was optimized (Supplemental Table S1) and synthesized by Kingsley Biotechnology Co., Ltd., in accordance with the codon preference of *E. coli*. Subsequently, the sequence was integrated into the pET-20b vector utilizing the *Nde*I and *Xho*I sites, resulting in the generation of pET20b-rhFEB. The recombinant plasmid pET20b-rhFEB was introduced into *E. coli* BL21(DE3), followed by screening for positive transformants on LB medium with 100 µg/mL ampicillin.

The strains were cultured in 5 mL of LB media at 37 °C until an optical density of 600 nm (OD_600_) between 0.8 and 1.0 was reached. Subsequently, the strains were induced with 1 mM isopropylthio-β-D-galactoside (IPTG) for 3.5 h. The expression of rhFEB was monitored via SDS‒PAGE. The high-expression strain was then cultured overnight in 50 mL of LB medium at 37 °C, after which 5 mL of the culture was added to 500 mL of LB medium. The cultures were grown at 37 °C for approximately 3 h and induced with 1 mM IPTG for 3.5 h. Biomass was obtained through centrifugation at a temperature of 4 °C. Subsequently, the purification of rhFEB was carried out using Ni column affinity chromatography and Sephadex G25 gel filtration. The purified samples were then subjected to analysis via SDS‒PAGE and Western blotting (WB), and their purity was assessed via HPLC.

### Cell viability assay

HaCaT cells were cultured in 96-well plates (4.0 × 10^3^ cells/well) for 15–18 h at 37 °C and 5% CO_2_. The suspended cells were removed by three rinses with PBS and the remaining cells were treated with 100 µL of tissue culture containing rhFEB (31.3 nmol/L, 62.5 nmol/L, 125.0 nmol/L, 250.0 nmol/L, 500.0 nmol/L) for 24 h and 48 h. After 10 µL of MTT was applied for another 4 h, the tissue culture was removed and the process was terminated with 100 µL of DMSO. Cell viability was quantified using a microplate reader at 570 nm, with 630 nm serving as the reference wavelength.

### Cell adhesion assay

Ninety-six-well plates were coated with 50 µL of either 31.3 nmol/L or 125 nmol/L rhFEB, or 125 nmol/L EGF as a positive control and ddH_2_O as a normal control. Next, the plates were washed three times with PBS. One hundred microliters of tissue culture mixture containing 1.0 × 10^4^ HaCaT cells was seeded onto the treated plates, and the cell were allowed to attach for 4 h at 37 °C and 5% CO_2_. After incubation, the nonadhesive cells were removed by three rinses with PBS. The remaining cells were incubated with fresh tissue culture containing 10 µL of MTT for another 4 h. The tissue culture was subsequently removed, and the process was terminated with 100 µL of DMSO. Adhesive cells were quantified using a microplate reader at 570 nm, with 630 nm as the reference wavelength.

### Cell migration assay

Cell migration was assessed using an in vitro scratch wound healing model. HaCaT cells were cultured in 12-well plates at a density of 1.0 × 10^5^ cells/well until they reached 95% confluence at 37 °C and 5% CO_2_. A scratch was created using a 200-µL pipette tip, and nonadhesive cells were removed using PBS. Subsequently, the cells were treated with 1 mL of sample containing either 31.3 nmol/L or 125 nmol/L rhFEB, or 125 nmol/L EGF in DMEM with 1% FBS. Images of the scratch area were captured using an inverted microscope at 0 h and 48 h. ImageJ software was used to determine percentage closure (%).

### Tube formation assay

A tube formation assay was conducted using ECV304-eGFP cells. Forty microliter samples (31.3 nmol/L or 125 nmol/L rhFEB or 125 nmol/L EGF in RPMI-1640 medium without FBS) mixed with 10 µL of Matrigel were incubated in precooled 96-well plates for 2 h at 4 °C, followed by solidification at 37 °C for 16 h. Subsequently, 100 µL of culture containing 1.5 × 10^4^ ECV304-eGFP cells was added to the wells and incubated at 37 °C and 5% CO^2^ for 12 h. The tubular structures were observed and captured with an inverted microscope (Olympus IX70, Tokyo, Japan). And the number of tubular structures was measured by Image J software.

### COL1A1 secretion assay

A COL1A1 secretion assay was performed with fibroblasts in vitro. Two milliliters of culture containing 6.0 × 10^4^ HSF cells was inoculated in 6-well plates at 37 °C and 5% CO_2_ until the cell confluence reached 45 ~ 60%. The culture was removed and the cells were treated with 2 mL of rhFEB (2 nmol/L, 20 nmol/L, 200 nmol/L), TGFβ1 (200 nmol/L) as the positive control, and DMEM without FBS as the normal control. After 24 h of incubation at 37 °C and 5% CO_2_, the supernatant of the cell culture was collected, and the COL1A1 concentration was subsequently measured via WB analysis.

### Western blot analysis

Cells or tissues were lysed in ice-cold RIPA lysis buffer and centrifuged for 15 min at 15,000 × g. Insoluble debris was removed from the supernatant lysate, and a protease inhibitor cocktail was added. The protein concentration was determined using a BCA protein assay (Thermo Scientific, Waltham MA). Total protein in equal amounts was separated via SDS‒PAGE and transferred onto nitrocellulose membranes. After the nonspecific binding was blocked using 5% fat-free milk in 0.1 M TBST, the membranes were incubated with primary antibodies against COL1A1 (1:1000; CST, #72,026) and GAPDH (1:5000; Bioworld, AMM04690G) overnight at 4 °C. The membranes were then washed five times with 0.1 M TBST, followed by incubation with horseradish peroxidase (HRP)–conjugated secondary antibodies for 1 h at room temperature. The blots were developed and assessed using Pierce ECL chemiluminescent substrates (Thermo Scientific, Waltham, MA).

### Expression of M1-type macrophage markers

Primary precursor cells were obtained and cultured from the bone marrow of male C57BL/6 mice (6–8 weeks, 20–25 g). These cells were then stimulated to differentiate into monocyte-derived macrophages with 25 ng/mL macrophage colony–stimulating factor (M-CSF1) and cultured in 10% FBS-DMEM for 7 days. Then, differentiated macrophages were exposed to inducers of M1 macrophages (100 ng/mL LPS and 20 ng/mL IFN-γ) for 24 h, followed by stimulation with rhFEB at concentrations of 31.3 nmol/L and 125.0 nmol/L for an additional 24 h. Total RNA was extracted from cells using a HiPure Fibrous RNA Plus Kit (Magen, Guangzhou, China) and was subsequently transcribed into cDNA via a reverse transcription kit (Tiangen Biotech, Beijing, China). qRT‒PCR was subsequently performed with a SYBR-Green Quantitative PCR Kit (Bio-Rad, Hercules, CA, USA) and the CFX96 Touch Real-Time PCR Detection System (Bio-Rad, Hercules, CA, USA). Relative expression of *IL-1β*, *TNF-α*, *CD206*, and *Arg-1* was quantified by the 2^−ΔΔCt^ method, with GAPDH serving as the reference gene.

### Full-thickness skin defect model in diabetes mice

Female mice with diabetes and a weight of 40 ± 2 g were administered anesthesia with 3% pentobarbital sodium. Depilatories were applied to remove hair from an approximately 3 cm × 3 cm area on the back of the mice. A skin wound, measuring approximately 0.8 cm^2^ and reaching the fascia, was then created using a 10-mm diameter corneal trephine. This wound was located approximately 2 mm below the scapula and on both sides of the midline of the spine. Following the modeling procedure, 0.1 g of rhFEB gel complex (50 nmol/L, 200 nmol/L) was evenly applied to the wounds of the mice, and the EGF gel complex (125 nmol/L) was used as a positive control. The basic gel complex consisting of 14% Poloxamer 407 and 6% Poloxamer 188 and ddH2O was used as model. The day of the wound incision was designated day 0, and subsequently, the dimensions and healing progress of the wounds were assessed and documented at 7, 10, 14, 21, and 28 days. Subsequently, the mice were humanely euthanized, and a section of normal skin approximately 3–4 mm from the wound edge was excised for further analysis.

### Histological and immunohistochemical staining

The skin tissue of the mice was collected and preserved overnight at a temperature of 4 °C in a 4% paraformaldehyde solution. Subsequently, the tissue was rinsed with water and subjected to dehydration using ethanol and xylene. The dehydrated tissue was then embedded in paraffin and sliced into thin sections measuring 5 µm using a microtome. These sections were subsequently placed onto glass slides and dried at 65 °C. Hematoxylin–eosin staining (H&E) and Masson trichrome staining were performed to analyze the tissue morphology of the repaired wounds. Immunofluorescence analysis was performed to stain the nuclei using DAPI (Beyotime, C1005), rabbit anti-mouse iNOS primary antibody (Proteintech, 80,517–1-RR) and FITC-labeled anti-rabbit secondary antibody (Servicebio, China) to label M1 macrophages. For immunohistochemical (IHC) staining, the slices were immersed in xylene twice for 5 min followed soaked in 100–70% ethanol for 3 min, and subsequently washed in PBS 3 times for 3 min. Citric acid buffer was used for antigen repair by boiling for 8 min and holding for 8 min, followed by natural cooling to room temperature, and rinsing with PBS 3 times for 3 min. Endogenous peroxidase activity was blocked by adding 3% H_2_O_2_ for 10 min in the dark, followed by blocking with 3% BSA-PBS for 30 min at room temperature. The slices were covered with CD31 Polyclonal antibody (1:500, Proteintech, 28,083–1-AP) at 4 °C overnight, followed by incubation with a goat anti-rabbit IgG secondary antibody (Gene Tech, #GK500705) for 30 min. After incubation, the slices were rinsed with PBS 3 times for 3 min and a fresh DAB chromogenic agent (Gene Tech, #GK500705) was added dropwise. The color development was controlled using a microscope and terminated by rinsing with water. After hematoxylin staining, the slices were dehydrated in 70–100% ethanol for 3 min and xylene twice for 10 min. The slices were sealed with neutral resin and observed with an inverted microscope (Olympus IX70, Tokyo, Japan).

### Statistical analysis

All data are represented by Mean ± SEM, and statistical analysis was conducted using GraphPad Prism 9.0 software. *T*-tests or one-way ANOVA was used to analyze the differences. * *P* < 0.05 indicates a statistically significant difference (*n* ≥ 3).

## Results

### Expression and purification of rhFEB

**Fig. 1:**
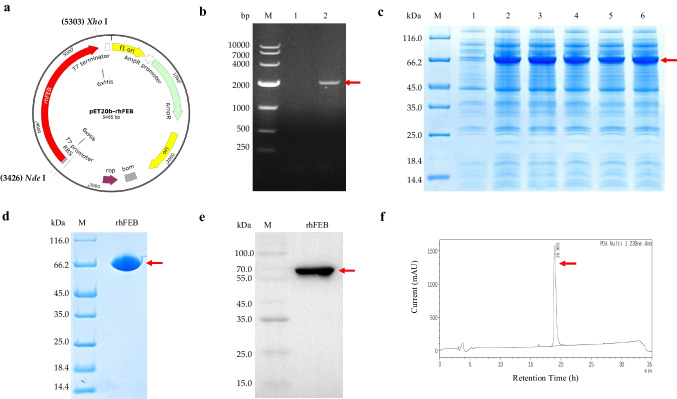
Expression and purification of rhFEB protein. **a** Construction schematic of the recombinant pET20b-rhFEB plasmid. **b** Nucleic acid electrophoresis of recombinant plasmid rhFEB. M: DNA Ladder 10,000; lane 1: negative control; lane 2: the bands of rhFEB were amplified by PCR. **c** Electrophoretic expression of rhFEB plasmid in BL21 (DE3) by SDS-PAGE; M: 14.4–116.0 kDa protein marker; lane 1: sediment of rhFEB before induction; lanes 2–6: sediment of different BL21 (DE3)/rhFEB monoclonal after induction. **d** Electrophoretic diagram of purified rhEGF protein; M: 14.4–116.0 kDa protein marker. **e** Western blotting analysis of recombinant rhFEB. M: 10–180 kDa protein marker. **f** HPLC analysis of the purity of rhFEB protein

The coding sequence of the rhFEB fusion (1869 bp) was inserted into the pET-20b plasmid distal to the T7 promoter. The resulting recombinant plasmid, pET20b-rhFEB, contains a 6-histidine tag for facilitating protein purification (Fig. 1a and b). The rhFEB protein was expressed in *E. coli* BL21 (DE3) through recombinant techniques, and induction was achieved by IPTG. Upon induction, the rhFEB protein exhibited a significant difference in expression compared to that in the control group, in which the protein had an approximate molecular weight of 60 kDa (Fig. 1c). The rhFEB protein was acquired using Ni affinity chromatography in a 100 mM imidazole eluant, followed by purification through a molecular sieve to obtain the desalted product. The verification of rhFEB expression and purification were conducted via Western blot analysis utilizing an anti-rhFEB monoclonal antibody (Fig. 1d and e).

### Cell biological viability of rhFEB on adhesion, migration, and tube formation

**Fig. 2:**
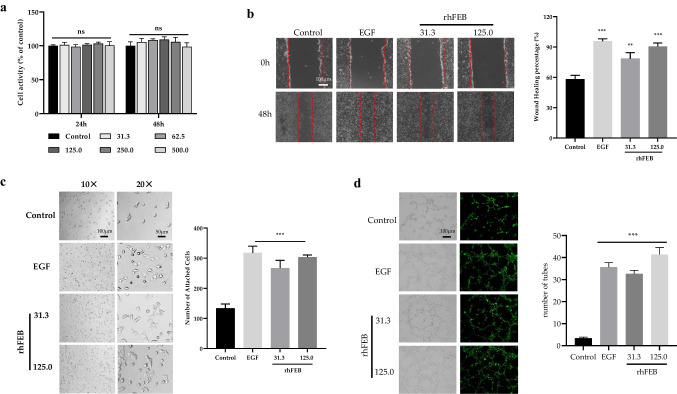
Cell biological visibility of rhFEB on proliferation, adhesion, and migration. **a** Proliferation of rhFEB to HaCaT cells. **b** Migration and healing rates of HaCaT cells by rhFEB (scale bar = 100 μm). **c** Adhesion of rhFEB to HaCaT cells (scale bar = 100 μm). **d** rhFEB-induced tube formation of ECV304-eGFP cells. *n* = 3, mean ± SD, ** *P* < 0.01, *** *P* < 0.001 vs. control group

HaCaT cells were used as a cellular model to examine the biological efficacy of rhFEB in terms of cell adhesion and migration, and the growth factor EGF was utilized as a positive control. Initially, a viability assessment of rhFEB demonstrated that concentrations ranging of 31.3–500 nmol/L for a duration of 48 h did not induce any toxicity in HaCaT cells (Fig. 2a). After 48 h of rhFEB or EGF administration, the wounds in the wound healing assay exhibited nearly complete closure (Fig. 2b). The wound healing rates of the rhFEB group at concentrations of 31.3 nmol/L and 125.0 nmol/L were 78.44% and 90.53% respectively, which were significantly greater than those of the control group, in which the healing rate was 58.02% (*P* < 0.001) (Fig. 2b). Additionally, the adhesion assay results revealed that the cells treated with rhFEB exhibited a spread-out morphology, whereas the control group exhibited a circular morphology (Fig. 2c). Moreover, compared with the control group, which had 133 adherent cells, the rhFEB group demonstrated a substantial increase in cell adhesion: 267 cells adhered at a concentration of 31.3 nmol/L, and 303 cells adhered at a concentration of 125.0 nmol/L (*P* < 0.001) (Fig. 2c). Additionally, the rhFEB protein displayed a remarkable ability to promote vascularization in the tube formation assay (Fig. 2d).

### rhFEB promoted COL1A1 secretion by HSF cells

**Fig. 3:**
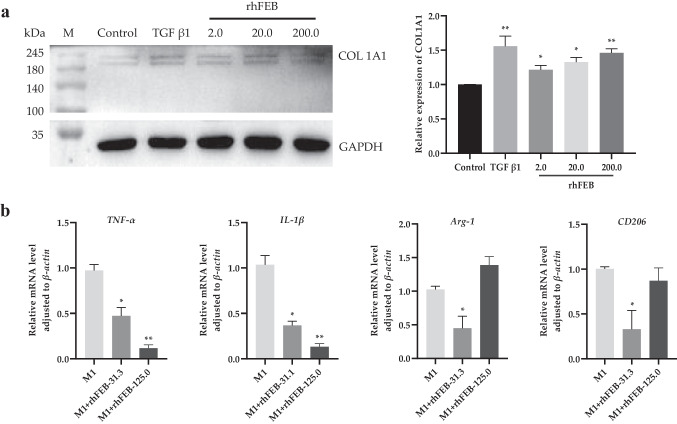
Cell biological activity of rhFEB on COL1A1 secretion and inflammatory reaction. **a** Expression of COL1A1 protein in HSF cells treated with TGFβ1 and rhFEB. **b** Expression of inflammatory markers, *TNF-α*, *IL-1β*, *Arg-1*, and *CD206* in M1 macrophage treated with rhFEB protein. *n* = 3, * *P* < 0.05, ** *P* < 0.01 vs. control group

The protein COL1A1 is the predominant elastic collagen protein found in the human body, and its intricate network architecture serves to safeguard and maintain the elasticity of the skin. With inadequate production or excessive degradation of COL1A1 within the dermis, the skin’s elasticity diminishes, resulting in signs of aging such as wrinkling and sagging. To investigate the impact of the recombinant rhFEB protein on the synthesis or release of COL1A1, we conducted in vitro experiments using fibroblasts. COL1A1 expression increased significantly in response to varying concentrations of rhFEB (2.0 nmol/L, 20.0 nmol/L, and 200.0 nmol/L), with corresponding fold changes of 1.2, 1.3, and 1.5, respectively, compared to that in the control group (*P* < 0.05) (Fig. 3a).

### rhFEB suppressed M1-type macrophage phenotypic differentiation

The expression of proinflammatory and anti-inflammatory factors is a typical characteristic of the inflammatory response, while alternative polarization typically leads to the expression of anti-inflammatory factors. Consequently, the gene expression of *TNF-α*, *IL-1β*, *Arg-1*, and *CD206* was used as a representative measure of macrophage phenotype. Compared to the normal group (M_0_), the expression levels of *TNF-α* and *IL-1β* in M1 group were significantly increased (*P* < 0.01) (Supplemental Figure S1). After treatment with rhFEB for 24 h, the expression levels of the *TNF-α* and *IL-1β* in M1 macrophage were rapidly decreased (Fig. 3b). However, the expression levels of *Arg-1* and *CD206* were inhibited with the treatment of low concentration rhFEB and restored to the original levels when treated with higher concentration rhFEB (Fig. 3b). These results suggested that the rhFEB has capacity of inhibiting the expression of inflammatory factors but not able to promote the polarization from M1 to M2.

### rhFEB improved wound healing of diabetes mice with full-thickness skin defects

**Fig. 4:**
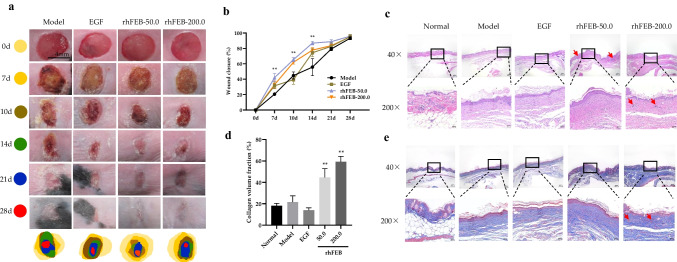
Effect of rhFEB on the skin wound healing. **a** Traces of wound closure were photographed at 0, 7, 10, 14, 21, and 28 days post-surgery. **b** Quantitative analysis of epithelial thickness at days 3, 7, 10, 14, 21, and 28 using ImageJ software. **c** H&E staining of wound sections at 28 days post-surgery (40 × , 200 ×). **d** and **e** Masson trichrome staining of wound sites at 28 days post-surgery(40 × , 200 ×). Arrows indicate hair follicle structure. *n* = 3, ** *P* < 0.01 vs. normal group

The process of wound healing is a complex and well-coordinated phenomenon that occurs during tissue regeneration. To assess the impact of rhFEB on diabetic wounds, we generated a full-thickness skin defect model in vivo. The evaluation was based on the wound healing time and wound area measurements within a predetermined time interval. Over time, wound contraction from the margins was observed in all the experimental groups, with db/db mice treated with varying concentrations of rhFEB exhibiting faster wound area contraction than did the model group mice at 7, 10, and 14 days. Notably, the db/db mice treated with rhFEB exhibited significantly accelerated wound closure on the 14th day following surgery. However, the wounds observed in the model group still exhibited greater severity than those in the rhFEB group on the 14th day. Subsequently, the wound exhibited ongoing healing progress, and during the period from day 14 to day 28 in the advanced phase of wound healing, the speed and area of wound healing tended to remain consistent across all groups of mice. Notably, all wounds in mice achieved a healing area exceeding 95% by the 28th day (Fig. 4a and b).

### Histopathological analysis

**Fig. 5:**
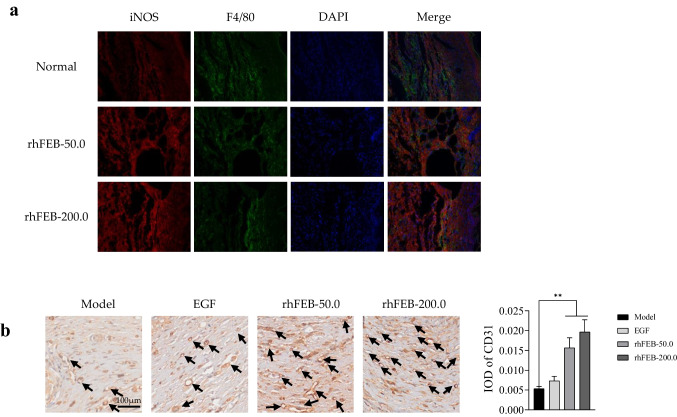
Inflammation and immunolabelling. **a** Inflammation labeling of iNOS positive cells 28 days after treatment. **b** The immunohistochemical staining of CD31 within the wounds at 28 days post-surgery. Arrows indicate CD31 expression (scale bar = 100 μm). ** *P* < 0.01 vs. model group

Wound healing involves various cellular interactions, encompassing inflammatory cells, keratinocytes, and fibroblasts. This intricate process entails cell migration, proliferation, and differentiation, ultimately resulting in angiogenesis, collagen deposition, ECM formation, re-epithelialization, and the formation of sebaceous glands and hair follicles (Hu et al. 2018; Zhao et al. 2017). To further investigate the effect of rhFEB on diabetic wounds in vivo, we performed histopathological analysis through H&E staining and Masson trichrome staining. H&E staining analysis, conducted 28 days after the defect was initiated, revealed the presence of an intact skin stratum corneum with a basal layer in all experimental groups. In comparison to those in the model group, the repaired tissue in the rhFEB treatment group exhibited normal hair follicle structures, as indicated by the arrow. Additionally, the H&E staining results revealed greater granulation and vascular ring structures in the skin tissue of the rhFEB groups following wound healing, than in that of the model group (Fig. 4c). Masson’s trichrome staining serves as a valuable tool for evaluating the comprehensive integrity of the scar region through the quantification of collagen deposition and morphology. Masson’s trichrome staining revealed that the application of rhFEB to diabetic wounds resulted in the deposition of ECM on day 28, particularly in relation to the blue-stained collagen. Moreover, the collagen volume fractions in the rhFEB group reached 44.57% and 59.23% at doses of 50 nmol/L and 200 nmol/L, respectively, which were significantly greater than those observed in the normal and model groups. Furthermore, the application of rhFEB notably enhanced the organization and density of collagen fibers on the treated wound surfaces (Fig. 4d and e). Neovascularization, a crucial aspect of wound healing, was assessed using CD31, a widely accepted marker for angiogenesis. The rhFEB-50.0 and rhFEB-200.0 groups exhibited significantly treater CD31 expression than the normal group (Fig. 5).

## Discussion

Diabetic wounds are typical chronic wounds that can cause severe problems in diabetic patients. Chronic wounds are characterized by pathological inflammation, and persistent inflammation is an inhibitory factor of diabetic wound healing (Louiselle et al. 2021). Wound healing involves four distinct stages: hemostasis, inflammation, proliferation, and tissue remodeling. Inflammation, which follows hemostasis, involves the phagocytosis and elimination of pathogens by immune cells. During the proliferative phase, the formation of blood vessels, granulation, and ECM scaffolds are synergistically stimulated by growth factors. Subsequently, re-epithelialization and collagen deposition progress during tissue remodeling, and vascular density return to low levels (Campos et al. 2008; Malone-Povolny et al. 2019).

The ECM serves as a structural framework that facilitates cellular functional expression and possesses regenerative and reparative capabilities (Yang et al. 2021). FNs and their subtypes, which are functional glycoproteins in the ECM, are crucial for cell–matrix interactions (Lenselink 2015; Parisi et al. 2020). Following injury, FN undergoes alternative splicing to produce an isoform known as EDB-FN (Natal et al. 2006). EDB-FN is involved in endothelial cell proliferation and angiogenesis (Khan et al. 2005). Collagen is implicated in the complete wound healing process. During the early stages, breakdown products of collagen can activate immune cells and facilitate inflammatory reactions. As wound healing progresses, collagen contributes to vascular regeneration, keratinocyte migration, collagen deposition, hair follicle development, and re-epithelialization (Elbialy et al. 2020; Lai et al. 2020).

Wound healing is closely related to the synergistic effect of FN and collagen. FN contacts collagen to activate variable internal signaling pathways to maintain ECM stability (Dalton and Lemmon 2021; Singh et al. 2010). FNs actively participate in multiple stages of wound healing, including hemostasis, proliferation, and remodeling. Therefore, developing therapies that augment the healing potential of FN through the synthesis of FN peptides and the targeted utilization of growth factors is interesting (Llopis-Hernández et al. 2016; Martino et al. 2011). Therefore, in this study, the active fragment of cellular FN, EDB-FN, and the triple-helix fragment of COL3A1, which is involved in cell adhesion, were recombined and expressed in an *E. coli* system. Finally, recombinant FN with a molecular weight of 60 kDa was obtained.

Angiogenesis is essential for tissue regeneration and an important indicator of wound healing processes. The formation of fresh blood vessels is particularly important for chronic wound healing (Bauer et al. 2005). Research has indicated that the presence of the EDB-FN domain can enhance the expression of VEGF, thereby facilitating the proliferation of endothelial cells and angiogenesis (Khan et al. 2005). In this study, we simulated vascular differentiation of ECV304-eGFP cells in vitro, and the findings revealed that the induction of ECV304-eGFP cells by rhFEB resulted in the formation of well-defined and abundant lumen structures compared to those in the control group. Additionally, the efficacy of rhFEB was further validated through IHC analysis of tissues from diabetic rats with full-thickness skin defects.

Early initiation of effective treatment is crucial for ensuring stable and orderly wound repair and achieving early attainment of primary healing. FN is involved in the complete process of wound repair, especially in the early stages (Barrenas et al. 2019). To further demonstrate the effect of rhFEB on diabetic wounds, we established a full-thickness skin defect model in diabetic mice. During the initial 2-week period, compared with those undergoing natural healing, the mice treated with rhFEB exhibited significantly faster wound healing. Although chronic and refractory conditions are typical features of diabetic wounds (Louiselle et al. 2021), and the wound healing process in experimental mice lasts for 28 days, rhFEB could promote the initial stages of healing. Furthermore, H&E staining and Masson staining revealed that rhFEB notably enhanced the development of hair follicles and granulation structures, as well as collagen deposition and organized arrangement during the wound remodeling phase.

Inflammation is an important process in wound healing. Inhibiting the expression of proinflammatory factors secreted by M1 macrophages or promoting the differentiation of inflammatory M1 macrophages into anti-inflammatory M2 macrophages is a conventional method for monitoring the development of inflammation (Van den Bossche et al. 2016). M1 and M2 macrophages and their corresponding marker genes are commonly used to evaluate the phenotypic transformation of proinflammatory and anti-inflammatory effects. In vitro findings demonstrated that rhFEB effectively suppressed the transcription of the proinflammatory factor *TNF-α* and *IL-1β* in M1 macrophages. However, we did not detect an increase in the expression of the anti-inflammatory factors *Arg-1* or *CD206*, indicating the absence of M1 to M2 conversion. It may be necessary to increase the administration time. Additionally, these findings imply that rhFEB may play an important role in the early stage of inflammation, particularly in the healing of diabetic wounds where M1 macrophages persist in the wound bed (Van den Bossche et al. 2016).

COL1A1, the predominant elastic collagen protein in the human body, possesses essential mechanical characteristics that contribute to flexibility. In the proliferative phase of wound healing, FN identifies and attaches to COL3A1, thereby transforming it into COL1A1 (Beyeler et al. 2019). In vitro, we successfully evaluated the secretion of COL1A1 by stimulating fibroblasts with rhFEB. In the diabetic wound model, Masson trichrome staining revealed a greater collagen deposition score in the tissue repaired with rhFEB.

In conclusion, through recombination technology, we constructed a new recombinant FN construct containing the EDB-FN active fragment of cellular FN and the triple-helix fragment of COL3A1 that has cell adhesive activities. The impact of rhFEB on chronic and respiratory skin ulcer wound healing was evaluated through a diabetic wound model. We confirmed that rhFEB has the potential to increase the initial healing rate of diabetic wounds, potentially through its inhibitory effect on proinflammatory factors. In addition, rhFEB has been shown to stimulate the production of COL1A1 and to promote better wound tissue remodeling and re-epithelialization. The design of the rhFEB protein still has shortcomings, such as the risk of metal-binding activities with the His-rich sequences, which should be improved and optimized in subsequent experiments. However, the molecular mechanisms involved, specifically those pertaining to the regulatory effect of rhFEB on macrophages during the diabetic wound healing, require further research.

## Supplementary Information

Below is the link to the electronic supplementary material.

Supplementary file1 (PDF 223 KB)

## Data Availability

The raw data supporting the conclusion of this article will be made available by the authors, without undue reservation.
