# Supplementary material for: Recombinantly expressed rhFEB remodeled the skin defect of db/db mice
Source: Appl Microbiol Biotechnol. 2024 Jan 29;108:183. doi: 10.1007/s00253-024-13021-9 (PMC10824822; doi:10.1007/s00253-024-13021-9)
Supplement: Supplementary file 1 [file 253_2024_13021_MOESM1_ESM.pdf]

Journal: Applied Microbiology and Biotechnology

Title: Recombinantly expressed rhFEB remodeled the skin defect of db/db mice

Authors:
Xiaomin Li[1], Xinliang Mao[1], Jianhang Cong[2], Qirong Zhang[3], Wenjie Chen[1], Kunjun Yan[3], Yadong Huang[2,3], Dun Su[1*], Qi Xiang[2,3*]

Affiliated Address:
[1]Perfect Life and Health Institute Co., Ltd., Zhongshan, China
[2]Institute of Biomedicine and Guangdong Provincial Key Laboratory of Bioengineering Medicine, Jinan University, Guangzhou, China,
[3]Biopharmaceutical R&D Center, Jinan University, Guangzhou, China

***Correspondence:** Dun Su, yfzx25@perfect99.com; Qi Xiang, txiangqi@jnu.edu.cn;

File type: Supplementary information

Table S1 Nucleotide sequences of the codon-optimized rhFEB.

Name	Nucleotide sequence (5’→3’)
rhFEB	atgcatcaccaccatcaccatggtgagcgtggtgcaccgggttttcgtggtcctgcaggtccg<br>aacggtatcccgggtgaaaaaggtcctgctggtgaacgtggtgcaccaggcgaacgtggt<br>gctccaggttttcgtggccctgctggtccaaatggcattccgggtgaaaaaggtccagca<br>ggtgaacgtggtgctccgggtgaacgtggtgctccaggttttcgtggtccagctggtcca<br>aacggcattccgggtgaaaaaggtcctgctggtgaacgtggtgcaccgggtgaacgtggt<br>gcaccaggttttcgtggtccggctggtccaaacggtattccgggcgaaaaaggtccggca<br>ggtgaacgtggtgcacctcctggtgaacgtggtgctcctggtttccgcggtcctgctggt<br>ccaaacggcattccgggtgaaaaaggtccagcgggtgaacgtggtgctccaggtgaaacc<br>ggtgctcctggtctgaaaggtgaaaatggtctgccaggtgaaaacggtgcaccgggtcct<br>atgggtccacgtggtgctccaggtgaaggtgaacgtggcgctccgggttttcgtggtcca<br>gctggtccaaatggcattccgggtgaaaaaggtccagccggtgaacgtggtgctccaggt<br>gaacgtggtgccccaggtttccgtggtcctgctggtcctaacggcatcccaggcgagaaa<br>ggtccagcaggtgaacgtggtgctccaggtccggctggtccaaacggtattcctggcgaa<br>aaaggtgaacgtggtgcaccgggtttccgtggtcctgctggtccaaacggcattcctggt<br>gaaaaaggtccagctggtgaacgtggtgccccaggtgaacgtggtgcacctggtttccgt<br>ggtccggctggtcctaacggcattccaggtgaaaaaggtccagcaggcgaacgtggtgca<br>ccaggtccagcaggtccgaatggtatcccgggtgaaaaaggtcctgcaggtgaacgtggt<br>gcaccgcgtggtgatggtgaacgtggtgctccaggtttccgtggtccggcaggtcctaac<br>ggtattccgggtgaaaaaggtccagcaggtgaacgtggtgcacctggtgaacgtggtgct<br>cctggttttcgtggtccggcaggtcctaacggtatcccgggtgagaaaggtccagctggt<br>gaacgtggtgcacctggtgaacgtggtgcaccgggttttcgtggtccggcaggtcctaac<br>ggtattccaggtgaaaaaggtccagctggtgaacgtggtgcaccaggtcctccgggtgaa<br>aatggtaaacctggtgaaccgggtccgaaaggtgatgcaggttctcctggtgctccaggt<br>gaacgtggtgcgccaggttttcgtggtccggcaggtcctaacggcattcctggtgaaaaa<br>ggccctgcgggtgaacgtggtgctccacatcaccatcaccaccacggttccggctccgaa<br>ggctctgaaggtgaaggtggctctgaaggctctgaaggcgaaggtcaatctaccgtctcc<br>gacgtcccgcgtgacctggaagttgttgctgctaccccaacctccctgctgatctcttgg<br>gacgcaccggccgtgactgttcgttattaccgtatcacctacggtgagactggtggcaac<br>tctcctgtccaggaatttactgtccctggttctaaaagcaccgctacgattagcggtctg<br>aaaccgggtgttgattacactatcaccgtttacgcggtgactggtcgtggtgatagcccg<br>gcatcctctaaaccgatctctatcaactaccgtacggagattgacaaaccgcaccaccac<br>caccatcactaa

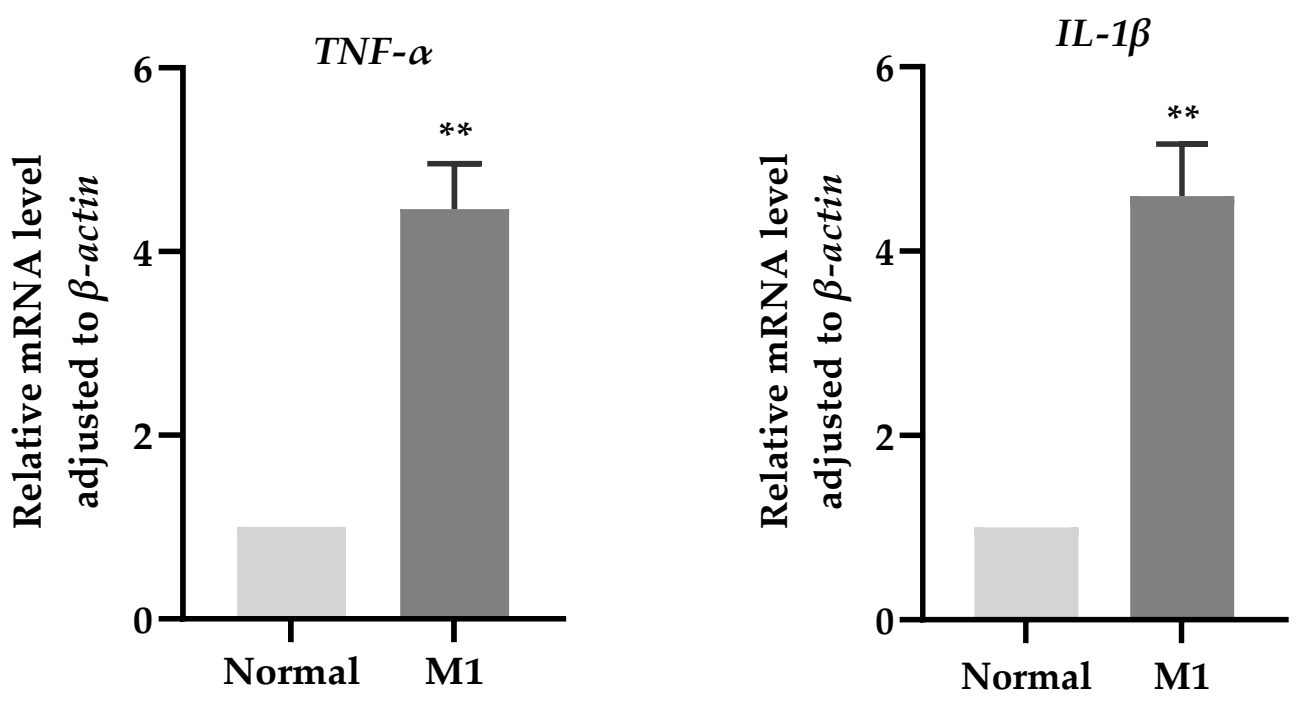

Fig. S1 Expression of inflammatory markers, *TNF-α* and *IL-1β* in M1 macrophage. n=3, ** $P < 0.01$ vs. normal.
